# Access to regulatory data from the European Medicines Agency: the times they are a-changing

**DOI:** 10.1186/2046-4053-1-50

**Published:** 2012-10-30

**Authors:** Beate Wieseler, Natalie McGauran, Michaela F Kerekes, Thomas Kaiser

**Affiliations:** 1 Institute for Quality and Efficiency in Health Care, Im Mediapark 8 (KölnTurm), Cologne, 50670, Germany

**Keywords:** Systematic reviews, Publication bias, Regulatory authorities, European Medicines Agency, Clinical study reports, Individual patient data listings, Raw data

## Abstract

Systematic reviewers are increasingly trying to obtain regulatory clinical study reports (CSRs) to correct for publication bias. For instance, our organization, the Institute for Quality and Efficiency in Health Care, routinely asks drug manufacturers to provide full CSRs of studies considered in health technology assessments. However, since cooperation is voluntary, CSRs are available only for a subset of studies analysed. In the case of the inhaled insulin Exubera, the manufacturer refused to cooperate and in 2007 we asked the European Medicines Agency (EMA) to provide the relevant CSRs, but EMA denied access. Other researchers have reported similar experiences.

In 2010 EMA introduced a new policy on access to regulatory documents, including CSRs, and has also undertaken further steps. The new policy has already borne fruit: in 2011, by providing additional sections of relevant CSRs, EMA made an important contribution to a review of oseltamivir (Tamiflu).

Unfortunately, speedy implementation of the new policy may be endangered. We define a CSR following the International Conference on Harmonisation (ICH) E3 guideline. Although this guideline requires individual patient data listings, it does not necessarily require that these listings be made available in a computer-readable format, as proposed by some regulators from EMA and other agencies. However, access to raw data in a computer-readable format poses additional problems; merging this issue with that of access to CSRs could hamper the relatively simple implementation of the EMA policy. Moreover, EMA plans to release CSRs only on request; we suggest making these documents routinely available on the EMA website.

Public access to regulatory data also carries potential risks. In our view, the issue of patient confidentiality has been largely resolved by current European legislation. The risk of other problems, such as conflicts of interest (CoIs) of independent researchers or quality issues can be reduced by transparency measures, such as the implementation of processes to evaluate CoIs and the publication of methods and protocols.

In conclusion, regulatory data are an indispensable source for systematic reviews. Because of EMA’s policy change, a milestone for data transparency in clinical research is within reach; let’s hope it is not unnecessarily delayed.

## Background

The effects of publication bias, i.e. the tendency to overestimate benefits and underestimate harms of health care interventions, are well known [1,2]. Some researchers from health technology assessment (HTA) agencies and other authors of systematic reviews therefore try to obtain full clinical study reports (CSRs) of all relevant studies in order to produce unbiased assessments. For example, our organization, the Institute for Quality and Efficiency in Health Care (IQWiG), which prepares HTA reports for the German statutory health care system, routinely asks the drug manufacturer to provide an overview of sponsored published and unpublished studies on the drug of interest. From this list we select the studies deemed relevant to the assessment and ask the manufacturer to submit the full CSRs. However, since submission of study overviews and CSRs by the manufacturer is voluntary, these documents are available only for a subset of the studies assessed (less than 40% [3]). For instance, in the case of the inhaled insulin Exubera, the manufacturer refused to cooperate and in March 2007 we therefore asked the European Medicines Agency (EMA) to provide the relevant CSRs. However, EMA denied access, stating that they needed the manufacturer’s consent to release the documents. Similarly, a request to EMA by two researchers from the Nordic Cochrane Centre, Gøtzsche and Jørgensen, to provide CSRs and protocols for two anti-obesity drugs was initially rejected in August 2007 [4]. EMA gave several reasons for not releasing the documents, among others, the protection of commercial interests. After a struggle of more than 3 years, including a complaint to the European ombudsman (who then publicly criticized EMA’s behaviour), the data were finally provided in February 2011.

## Main text

### Non-inclusion of clinical study reports leads to biased evidence syntheses

Previous research has shown that the non-inclusion of CSRs in systematic reviews and HTAs results in an incomplete evidence base and potentially biased conclusions about the effects of an intervention [3,5,6]. In an analysis of primary studies and corresponding documents (registry reports, CSRs, journal publications) from 16 HTAs of drugs conducted by IQWiG between 2006 and February 2011, we investigated to what extent these three types of documents deliver sufficient information for trial evaluation. The HTAs included 268 studies: publications, CSRs and registry reports were available for 192 (72%), 101 (38%), and 78 (29%) studies, respectively. Reporting quality was highest in the 101 CSRs, which overall provided complete information on 90% (1086/1212) of 12 required items for study methods and outcomes (e.g., reporting of randomization, allocation concealment, the primary endpoint, and adverse events). By contrast, registry reports and publications provided complete information on only 51% (477/936) and 46% (1052/2304) of items, respectively [3].

In 2009 IQWiG published an HTA report on reboxetine and other antidepressants [7]. The manufacturer only provided the relevant CSRs after an intense public debate. The inclusion of unpublished data from these CSRs (additional data on published studies as well as completely unpublished studies) called into question the previous positive conclusions about the benefits and harms of reboxetine based on published data alone. These data actually overestimated the benefits of reboxetine by up to 115% and 23% versus placebo and active comparators respectively, and also underestimated harms [5].

A further example is the case of the neuraminidase inhibitor oseltamivir (Tamiflu) experienced by Doshi, Jefferson and other colleagues, who were the authors of a Cochrane review on the drug. Claims concerning its effectiveness in reducing important complications of influenza were based on a meta-analysis of mostly unpublished industry-sponsored trials [8]. In September 2009, in an attempt to verify these claims, the authors requested the relevant data from the manufacturer, who at first unsuccessfully tried to implement a confidentiality agreement, and then provided data that were incomplete and insufficient to verify the methods and results of the unpublished trials [8]. After further attempts to obtain the relevant data, the manufacturer provided about 3200 pages of CSRs in 2010. Further tens of thousands of pages were provided by EMA in 2011 after a Freedom of Information request. The authors concluded that this unpublished information had “turned our understanding of the drug’s effects on its head” [6]. Among other things, the CSRs provided information that allowed the authors to determine the total number of trials that might fit the systematic review inclusion criteria, to analyse serious adverse events not mentioned in published papers, and to assess the validity of published information. However, the evidence on Tamiflu is still incomplete as the full set of CSRs in the manufacturer’s possession has not been released [6].

### First step to a solution: EMA changes its policy

As already indicated above, in 2010 EMA introduced a new policy on access to clinical trial documents, including the release of documents submitted as part of marketing authorization applications (e.g. CSRs), after procedures concerning a drug had been finalized [9]. The change in attitude by regulatory authorities has also been echoed in a recent article by European regulators from EMA and other agencies (Eichler et al.), who stated that it was “neither desirable nor realistic to maintain the status quo of limited availability of regulatory trials data” [10].

EMA has also recently taken further steps to put the new policy into practice by clarifying open issues raised in a public consultation. The agency has published an update of its policy document, as well as an accompanying guidance document on the handling of commercially confidential information and personal data [11,12].

## Discussion

### Differing definitions of clinical study reports

We define a CSR following the International Conference on Harmonisation (ICH) E3 guideline [13]. According to ICH E3, a CSR, in addition to containing the full protocol, a statistical analysis plan, and summarized efficacy and safety data on all outcomes, also contains individual patient data in the format of tabulations or listings, but not necessarily in the format of an electronic database ready for computer-based analysis. (This type of format is only intended to be made available by sponsors on request but is not a regular component of the CSR [13]). However, the above regulators extend this definition of a CSR to include the full raw data set, which on the basis of the suggested applications would have to be provided as an electronic database. It has long been proposed to make raw data from clinical trials available for re-analysis [14-17], and besides verifying the results of clinical trials or testing additional hypotheses, access to such data may also offer additional advantages, such as enabling the development of innovative strategies for the individualized management of patients [10].

### Speedy implementation of EMA policy needed, not complication

We acknowledge the potential advantages of analysing raw data and that making both CSRs and raw data in a computer-readable format publicly available in the near future would be a huge step forward. We thus understand the rationale for this proposal. However, compared to public access to “conventional” CSRs, public access to CSRs supplemented by raw data in a computer-readable format would require additional measures, for example, to prevent misuse in the form of selective reanalyses of data by other researchers. We fear that the discussions about these requirements and their implementation will lead to long and unnecessary delays. In our opinion, making full CSRs available and providing raw data in a computer-readable format are two separate issues. Merging them would hamper the relatively simple (but ground-breaking) implementation of the new EMA policy. Instead, the requirements for publishing such raw data should be discussed in parallel.

A further point to consider is that EMA plans to release CSRs only on request. We would suggest making these documents routinely available on the EMA website after marketing authorization. This could be done by including a link to the CSRs of studies considered in the authorization process and listed in the European Public Assessment Reports (EPAR).

### Risks and benefits of access to regulatory data

Patient confidentiality has been a major concern in the discussion about making extended information (including individual patient data) from clinical trials available. Since CSRs also include individual patient data listings, in principle, this issue not only applies to raw data in a computer-readable format but also to CSRs. However, as stated by EMA, “current European legislation requires patient information to be included in non-identifiable form in the marketing authorization application submitted to competent authorities” [12]. Given these requirements, it seems unrealistic that data listings in a CSR would contain patient-identifying information, although the risk might be higher for raw data sets, where patient characteristics can be re-arranged and combined electronically. As Eichler et al. note, there might be exceptional cases (for example, trials in “ultra-rare” diseases) where it could be difficult to ensure patient confidentiality. In such cases, a simple preliminary solution would be to split off individual patient data listings before the release of CSRs. This would allow discussion of measures to ensure patient confidentiality without delaying the release of large parts of the information on these trials.

Other potential problems, such as conflicts of interest (CoIs) of “independent” researchers and the misuse of public access to CSRs for personal or competitive purposes probably cannot be fully avoided. However, this also applies to systematic reviews based on journal publications. The risk of CoIs (and of quality deficits) in systematic reviews can be reduced by transparency measures. For example, organizations such as IQWiG and the Cochrane Collaboration publish their methods, protocols and reports online and have also implemented processes to evaluate CoIs of participating researchers [18,19]. It should be noted here that commercial CoIs, including data spinning and the withholding of study results, have demonstrably caused substantial damage [2]. Previous drug safety catastrophes, such as the cases of rofecoxib (Vioxx) and class 1 antiarrhythmic agents, which harmed tens of thousands of patients [4,20,21], highlight the need for access to CSRs beyond regulatory agencies. In addition, access to such documents could help avoid wasting public funds, as exemplified in the case of Tamiflu, where in the United States alone over a billion dollars were spent on stockpiling a drug that “may be no better than aspirin” [6]. Overall, in our view the benefits of public access to CSRs far outweigh the risks.

## Conclusion

In conclusion, regulatory data in the form of CSRs are an indispensable source for systematic reviews. The debate between EMA, its stakeholder groups and interested parties on access to CSRs, including the issue of the level and format of data to be provided, is still ongoing [22]. As a result of EMA’s policy change, a milestone for data transparency in clinical research is within our reach; let’s hope it is not unnecessarily delayed by an insistence that raw data in a computer-readable format be released together with CSRs.

## Competing interests

Non-financial competing interests. All authors are employees of the Institute for Quality and Efficiency in Health Care, Cologne, Germany. In order to produce unbiased health technology assessment reports, the Institute depends on access to all of the relevant data on the topic under investigation. The authors therefore support public access to clinical study reports.

## Authors' contributions

BW had the idea for the paper. NM drafted the first version, which BW, MFK and TK revised. All authors have seen and approved the final version.
